# Supplementary material for: Dynamic Changes in Metabolite Accumulation and the Transcriptome during Leaf Growth and Development in Eucommia ulmoides
Source: Int J Mol Sci. 2019 Aug 18;20(16):4030. doi: 10.3390/ijms20164030 (PMC6721751; doi:10.3390/ijms20164030)
Supplement: Supplementary file 1 [file ijms-20-04030-s001.zip › supplementary figures and table S1-S2, S7.pdf]

Supplementary Materials

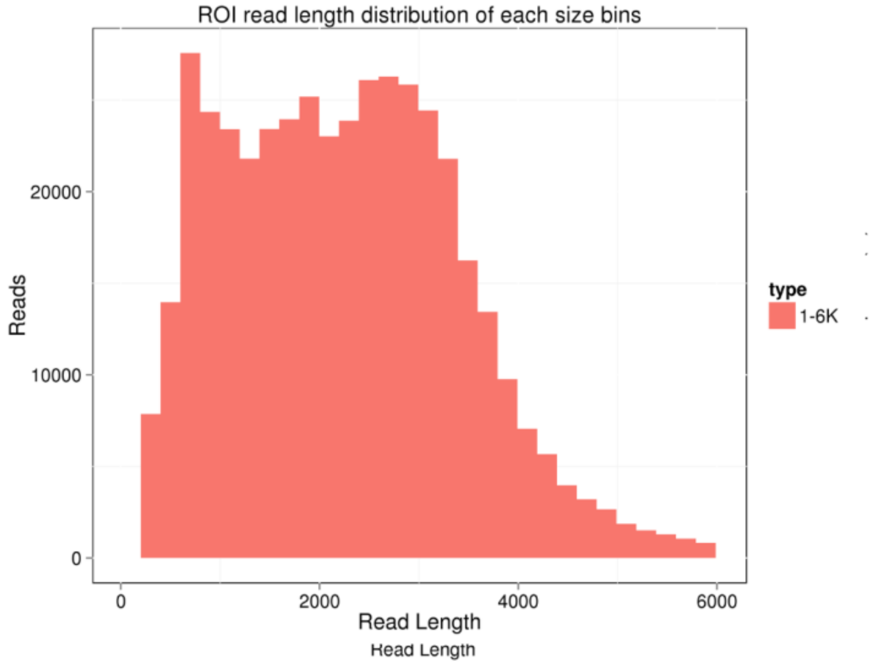

Figure S1. Distribution of read length in the F1 library.

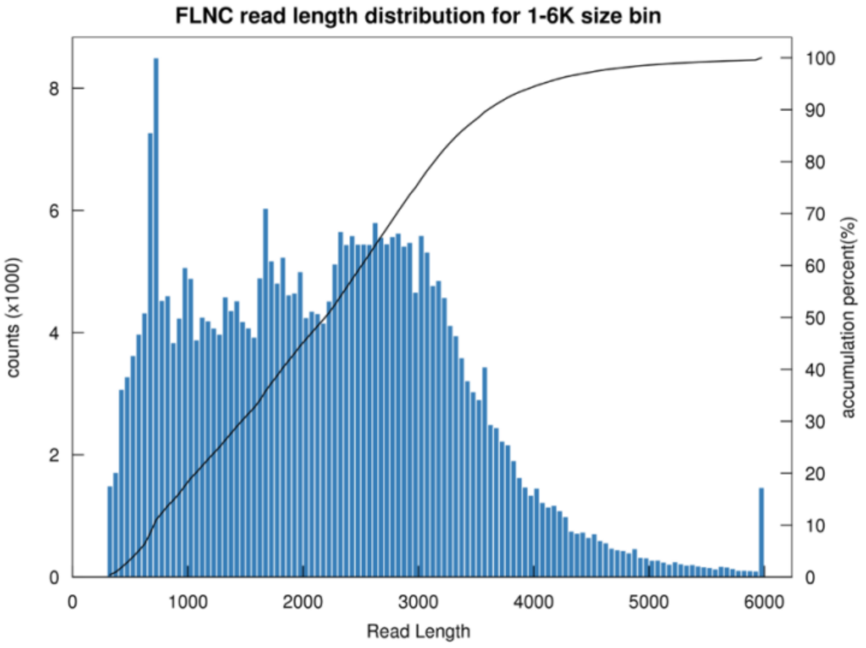

Figure S2. Distribution of read length of full-length non-chimeric (FLNC) reads in the single-molecule long-read sequencing library.

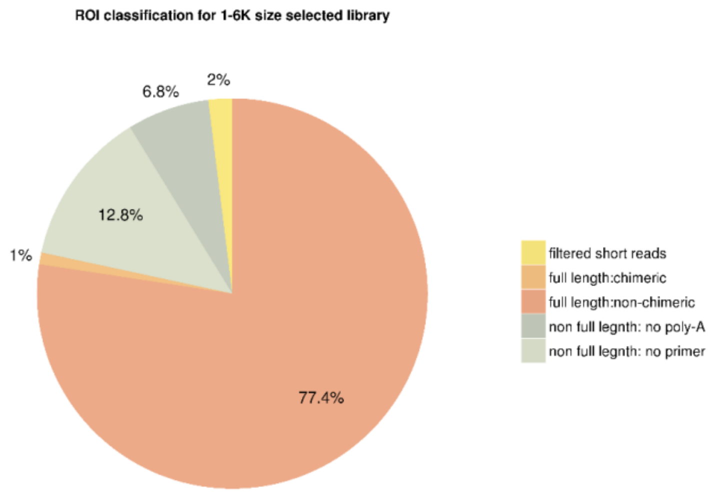

Figure S3. ROI classification for 1-6k size selected library.

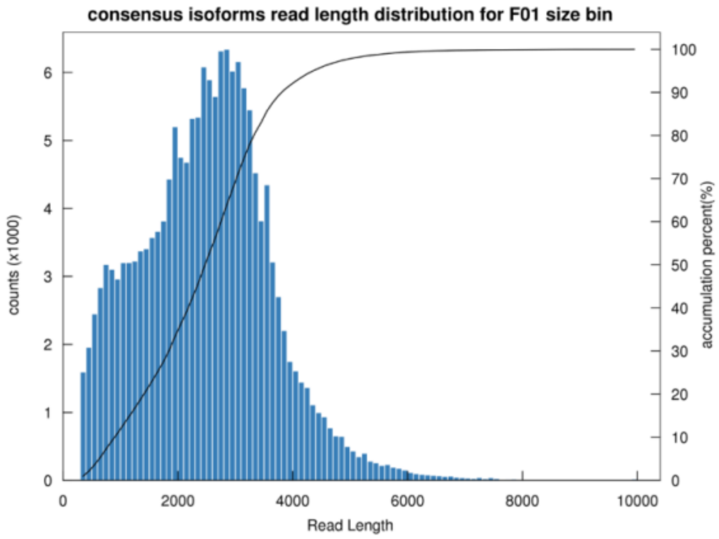

Figure S4. Distribution of read length of consensus isoforms reads in the single-molecule long-read sequencing library.

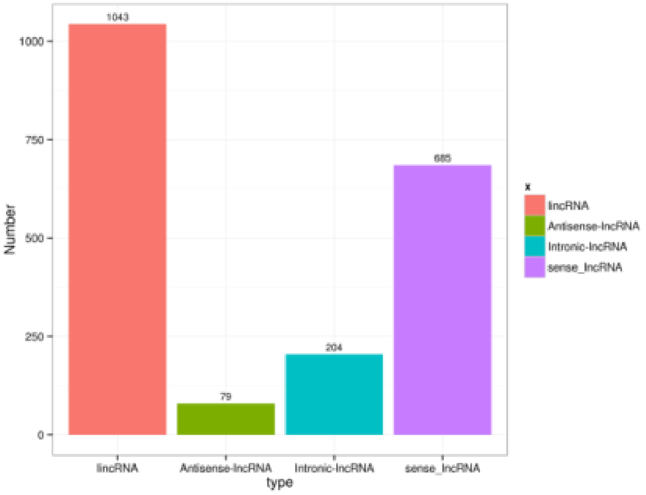

Figure S5. The quantity of each type IncRNA.

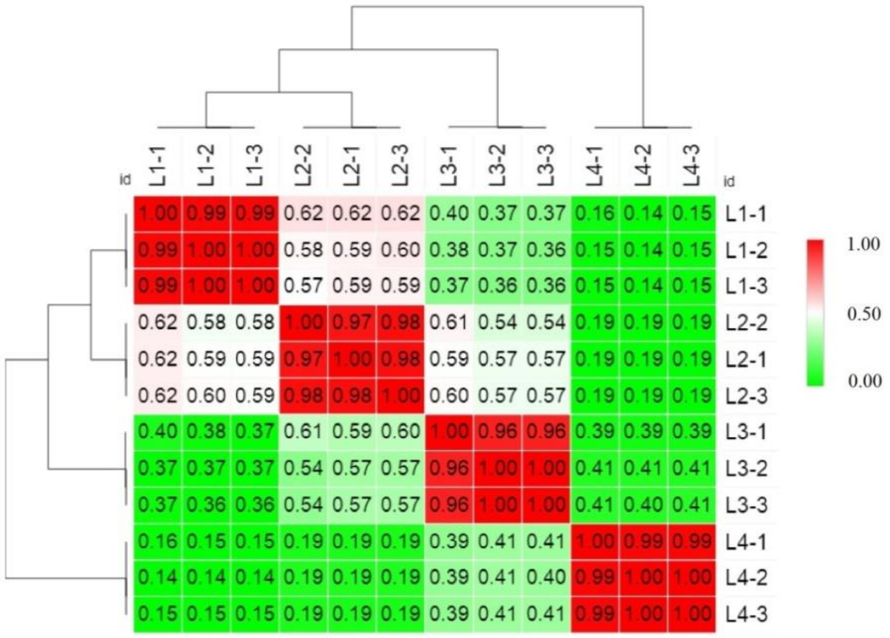

Figure S6. Hierarchical clustering of experimental samples based on isoform expression level.

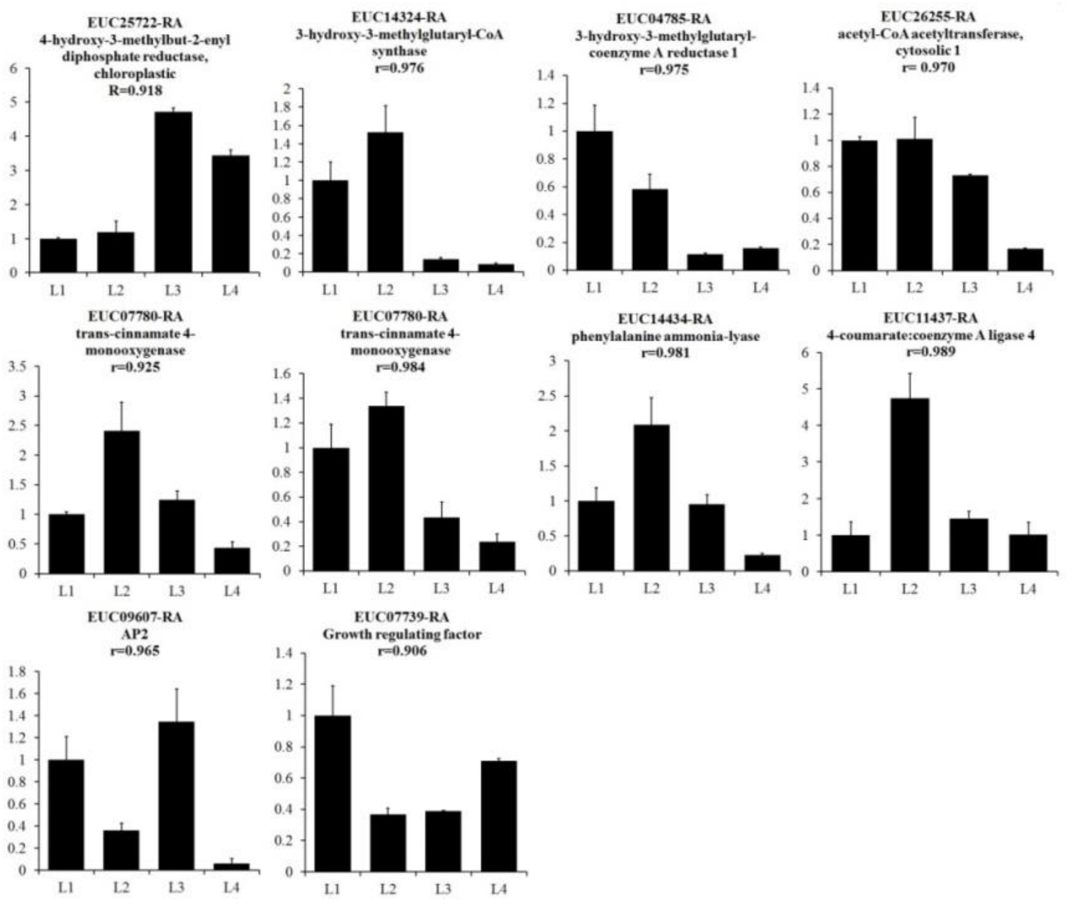

Figure S7. Verification of gene expression by qRT-PCR. The relative amount of mRNA (y-axis) is a ratio normalized by *Ubiquitin-conjugating enzyme E2* (*UBC E2*). The leaf growth stages are on the x-axis. R indicates the correlation coefficient for the expression between RNA-Seq and qRT-PCR data. The expression of each gene in L1 was arbitrarily set at 1.0.

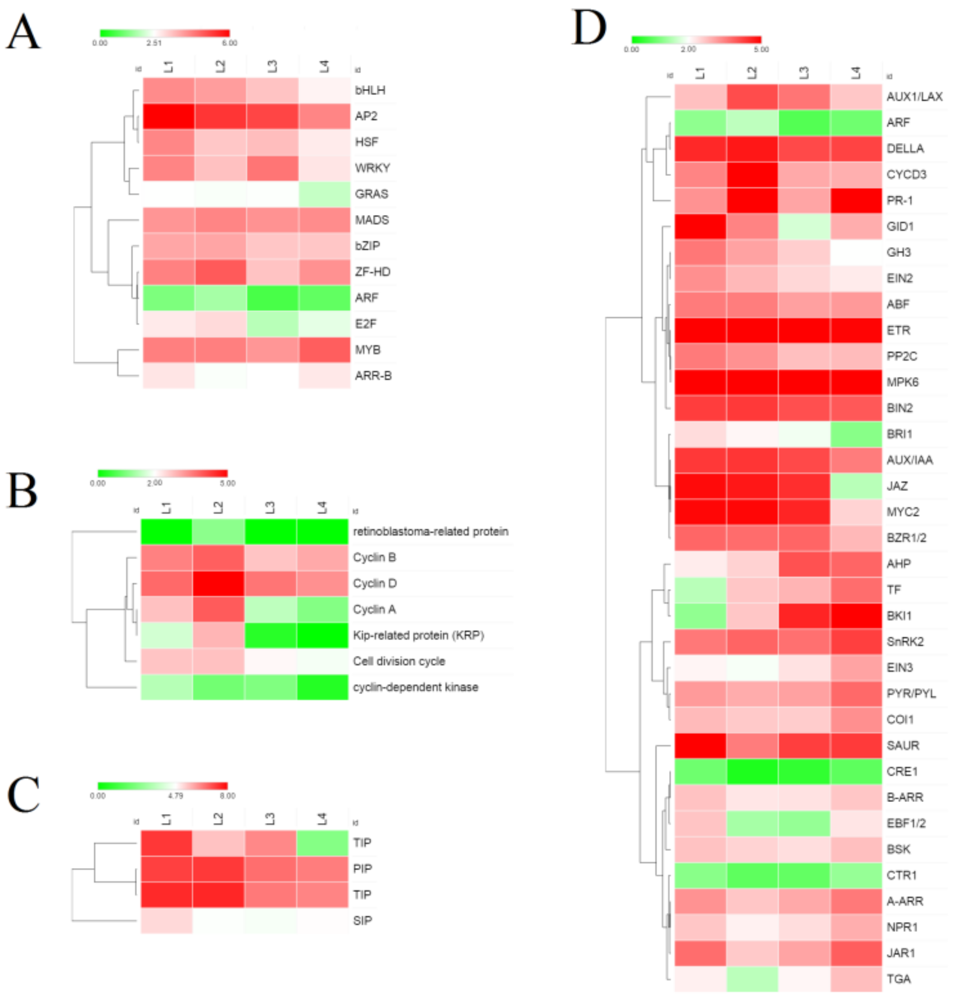

Figure S8. Expression profiles of isoforms involved in *E. ulmoides* leaf growth and development. (a) plant growth, flavonoid and Phenylalanine synthesis related transcription factor, (b) cell cycle, (c) aquaporins, (d) plant hormone signaling. Green indicates low expression, and red indicates high expression. The colour scale represents log_2-transformed FPKM values.

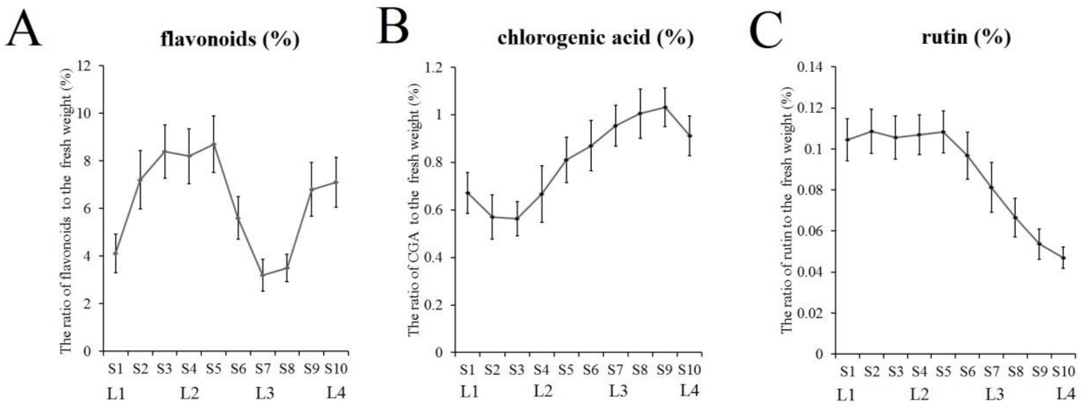

Figure S9. The ratio of flavonoids, chlorogenic acid (CGA) and rutin to fresh weight during leaf growth and development. (a) flavonoids, (b) chlorogenic acid, (c) rutin. The Y-axis represents the metabolite content ratio, while the X-axis represents the different leaf growth stages. The corresponding stages used for RNA-seq and metabolome profiling are shown below the abscissa.

Table S1. Reads of insert in each single-molecule long-read sequencing libraries.

Samples	cDNA Size	Reads of Insert	Number of five prime reads	Number of three prime reads	Number of poly-A reads	Number of filtered short reads	Number of non-full-length reads	Number of full-length reads	Number of full-length non-chimeric reads	Full-Length Percentage (FL%)	Artificial Concatemers（%）
F01	All	437,720	389,156	389,428	372,847	8,628	85,992	343,100	338,813	78.38%	1.25%

Table S2. Overview of mapping of the Second-generation sequencing reads.

Sample	Total Reads	Mapped reads	Uniquely mapped reads	Multiple mapped reads
L1-1	44176832	92.06%	84.72%	7.34%
L1-2	43119074	91.72%	85.02%	6.70%
L1-3	41524958	91.99%	85.46%	6.53%
L2-1	42440780	91.08%	83.29%	7.79%
L2-2	44752828	92.25%	81.98%	10.26%
L2-3	52310780	91.08%	83.14%	7.95%
L3-1	40981618	89.96%	78.64%	11.32%
L3-2	44865570	90.11%	83.58%	6.52%
L3-3	43083694	90.16%	83.41%	6.75%
L4-1	43664172	90.67%	84.02%	6.65%
L4-2	42693852	90.70%	83.58%	7.11%
L4-3	49195088	90.95%	84.45%	6.50%

Table S7. Primers used for qRT-PCR analysis.

Primer name	Annotation	Sequence
EUC07780-RA F<br>EUC07780-RA R	trans-cinnamate 4-monooxygenase	CACATGAACCTCCATGATGC<br>TAATTCCGAGAATCGGCAAG
EUC14434-RA F<br>EUC14434-RA R	phenylalanine ammonia-lyase	GCTGCGATCGGAAAACTCAT<br>TGAGATCAAGCCCAACGAGT
EUC04785-RA F<br>EUC04785-RA R	3-hydroxy-3-methylglutaryl-coenzyme A reductase 1	CTCTATGTTGTTGGCGGACG<br>GGAGACCATGTTCATCCCCA
EUC26255-RA F<br>EUC26255-RA R	acetyl-CoA acetyltransferase, cytosolic 1	TGTTGCACGTACACCAATGG<br>GCCCCAAATTTGCACTGAGA
EUC25722-RA F<br>EUC25722-RA R	4-hydroxy-3-methylbut-2-enyl diphosphate reductase, chloroplastic	GACTTGACTTTGCCGGAGAC<br>CGCGATTCATGAGCTCCAAA
EUC14324-RA F<br>EUC14324-RA R	3-hydroxy-3-methylglutaryl-CoA synthase	GACTGTGGACGCTGATGAGA<br>GAAAATTGTTTGCCCTCCAA
EUC09607-RA F<br>EUC09607-RA R	AP2	GGGAAGGCATTCGAAATACA<br>CGCGTAAGATGGCAACAAAT
EUC07739-RA F<br>EUC07739-RA R	Growth regulating factor	TCGGTTTTGGAGGCATTTGG<br>GGTCCCGGAATGCCCTATAA
EUC07780-RA F<br>EUC07780-RA R	trans-cinnamate 4-monooxygenase	CACATGAACCTCCATGATGC<br>TAATTCCGAGAATCGGCAAG
EUC11437-RA F<br>EUC11437-RA R	4-coumarate:coenzyme A ligase 4	GCACTGTTGGAGCAGATTCA<br>GGCAGTCTGGTACGAAGAGC
QUBCF<br>QUBCR	UBC E2	AGTGGGTGGTGCTGTAGTCC<br>AACTCCCGTTTCGTTTGTTG
AS EUC12362-RA F<br>AS EUC12362-RA R	bZIP	GCTTCAATTACTCGTTGATGCT<br>GGC

		GTGATAGAGCATCTTCTGCCTGCTG
AS EUC14737-RA F	Camellia sinensis mitoferrin-like	GCAACCTACGAGGCGGCAAA
AS EUC14737-RA R		ATCGGGAACCATTTACACGAACTCA
AS EUC14719-RA.gene F	PHD finger transcription factor	TGACAGTAGCGGTTATGGAGGTTC
AS EUC14719-RA.gene R		CACTAAGCAAAATTTAAGGTCCCTTCC
AS PB.1020 F1	RING/FYVE/PHD zinc finger superfamily protein	TCGATCGTGAACCCATCCATCGAAAC
AS PB.1020 R1		TCACATGACATGGAAGCCAAATATCT
AS PB.1020 F2	RING/FYVE/PHD zinc finger superfamily protein	GGGCTTTCAGTTTATTGCAGCGTTGAA
AS PB.1020 R2		GGTCCTGATTGTGCTCTTGTCATCGAT
